# Positive prognostic value of HER2-HER3 co-expression and p-mTOR in gastric cancer patients

**DOI:** 10.1186/s12885-017-3851-y

**Published:** 2017-12-12

**Authors:** Guo-dong Cao, Ke Chen, Bo Chen, Mao-ming Xiong

**Affiliations:** 1 Anhui Medical University, Hefei, Anhui 230022 China; 2 Department of General Surgery, First Affiliated Hospital of Anhui Medical University, Hefei, Anhui 230022 China

**Keywords:** HER2, HER3, mTOR, Prognosis, Gastric cancer

## Abstract

**Background:**

The HER2-HER3 heterodimer significantly decreases survival in breast cancer patients. However, the prognostic value of HER2-HER3 overexpression remains unknown in gastric cancer (GC).

**Methods:**

The expression levels of HER2, HER3, Akt, p-Akt, mTOR and p-mTOR were examined in specimens from 120 GC patients by immunohistochemistry and quantitative reverse transcription-PCR. The associations of HER proteins, PI3K/Akt/mTOR pathway-related proteins, clinicopathological features of GC, and overall survival (OS) were assessed. To comprehensively evaluate the prognostic values of pathway-related proteins, meta-analyses were conducted with STATA 11.0.

**Results:**

HER2 overexpression was significantly associated with HER3 levels (*P* = 0.02). HER3 was highly expressed in gastric cancer tissues. High HER2 and HER3 levels were associated with elevated p-Akt and p-mTOR amounts (*P* < 0.05). Furthermore, HER2-HER3 co-expression was associated with high p-Akt and p-mTOR (*P* < 0.05) levels. Meanwhile, p-mTOR overexpression was tightly associated with differentiation, depth of invasion, lymph node metastasis, TNM stage and OS (*P* < 0.05). By meta-analyses, Akt, p-Akt, and mTOR levels were unrelated to clinicopathological characters. HER3 overexpression was associated with depth of invasion (OR = 2.39, 95%CI 1.62–3.54, *P* < 0.001) and lymph node metastasis (OR = 2.35, 95%CI 1.34–4.11, *P* = 0.003). Further, p-mTOR overexpression was associated with patient age, tumor location, depth of invasion (OR = 1.63, 95%CI 1.08–2.45, *P* = 0.02) and TNM stage (OR = 1.73, 95%CI 1.29–2.32, *P* < 0.001). In addition, HER2-HER3 overexpression corresponded to gradually shortened 5-year OS (*P* < 0.05), and significant relationships were shown among HER3, p-mTOR overexpression, and 1-, 3-, 5-year OS (*P* < 0.05).

**Conclusions:**

HER2-HER3 co-expression may potentially enhance mTOR phosphorylation. HER2-HER3 co-expression and p-mTOR are both related to the prognosis of GC patients.

**Electronic supplementary material:**

The online version of this article (10.1186/s12885-017-3851-y) contains supplementary material, which is available to authorized users.

## Background

Gastric cancer (GC), one of the most frequently diagnosed malignancies, is also the leading cause of cancer-related death worldwide [1]. Surgical resection is the most effective treatment for GC, and the efficacy of chemotherapy remains limited [2]. The prognosis of patients with advanced GC remains dismal even after surgery or radical resection; 5–year overall survival (OS) is low, with a median OS of less than 1 year [3, 4].

In recent years, molecular-targeted treatment for GC has attracted increasing attention. Several articles have described potential molecular targets for GC therapy, such as epidermal growth factor receptor (EGFR) and vascular endothelial growth factor (VEGF) [5]. EGFR is a member of the human epidermal growth factor receptor (HER) family. The HER family is composed of four members, including EGFR/HER1/ErbB1, HER2/ErbB2, HER3/ErbB3, and HER4/ErbB4 [6], and plays a key role in the pathogenesis of various human solid tumors, including breast, gastric and lung cancers [7].

Overexpression of HER family members and their downstream signaling effectors demonstrates that these molecules play significant roles in the tumorigenesis, progression, chemotherapeutic resistance, and distant metastasis of various human cancers [8]. Of the four members, HER2 has no ligand, and the intrinsic tyrosine kinase domain of HER3 is defective and unable to form a homodimer [9]. Despite their individual limitations, HER3 contributes synergistically to HER2-mediated cell transformation and amplifies malignant properties of a tumor driven by HER2 overexpression; indeed, the HER2-HER3 heterodimer is considered the most potent HER mitogenic complex, which functions as an oncogenic unit that activates the phosphoinositide 3-kinase/protein kinase B (PI3K/Akt) and mitogen-activated protein kinase (MAPK) pathways in cancer [10–12]. In addition, some breast cancer patients show clinical benefits in HER2-amplified breast cancers through inhibition of HER2-HER3 dimer formation [13].

However, the clinicopathological and prognostic roles of the HER2-HER3 heterodimer in cancer remain controversial. Li et al. [14] found that HER2-HER3 co-expression leads to shorter survival in GC patients. This observation was concordant with findings obtained in extrahepatic cholangiocarcinoma (EHCC) [15]. However, in colorectal cancer, no significant relationship was found between HER2-HER3 co-expression and OS [16]. Moreover, few studies have investigated the activation mechanism of the PI3K/Akt/mTOR signaling pathway that is mediated by the HER2-HER3 heterodimer.

HER2-HER3 co-expression in GC is poorly understood. In this study, not only HER2 and HER3 expression, but also the levels of the PI3K/Akt/mTOR pathway-related proteins Akt, p-Akt, mTOR, and p-mTOR were assessed by IHC in 120 GC tissue samples. Meta-analyses were performed to further evaluate interlinks between HER family members and pathway-related proteins by comparing the consistency of prognostic significance. Our results suggested that HER2-HER3 co-expression leads to the phosphorylation of Akt and mTOR, which resulted in worse prognosis and shorter OS through a mechanism dependent on activated mTOR (p-mTOR).

## Methods

### Patients and samples

A total of 120 GC tissue samples were collected from patients who underwent total or partial gastrectomy at the First Affiliated Hospital of Anhui Medical University from 2010 to 2011, with no pre-operative chemo- or radiotherapy. Age, gender, tumor location and differentiation, depth of invasion, lymph node metastasis, distant metastasis, and TNM staging in patients were determined by reviewing their medical records. Tumor samples were classified according to the tumor–node–metastasis (TNM) classification system recommended by the International Union against Cancer [17]. Follow-up time was estimated from the date of surgical treatment to that of an event (i.e., patient death or tumor recurrence) or withdrawal. This study was approved by the local ethics committee of the First Affiliated Hospital of Anhui Medical University (The ethics approval documentation was uploaded as an Additional file 1).

### Immunohistochemistry

**Fig. 1:**
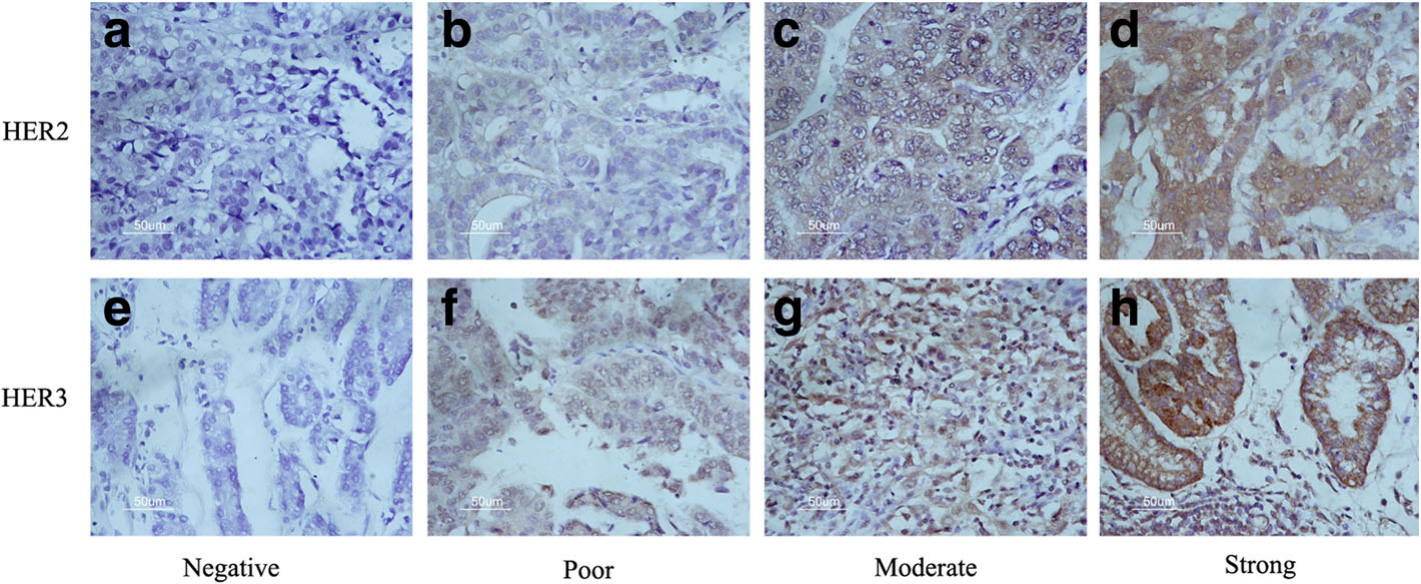
Immunohistochemical (IHC) staining of HER2 and HER3 expression in gastric cancer. HER2 negative staining (**a**), HER2 weak staining (**b**), HER2 moderate staining (**c**) and HER2 strong staining (**d**). HER3 negative staining (**e**), HER3 weak staining (**f**), HER3 moderate staining (**g**) and HER3 strong staining (**h**).Original magnification, ×200

**Fig. 2:**
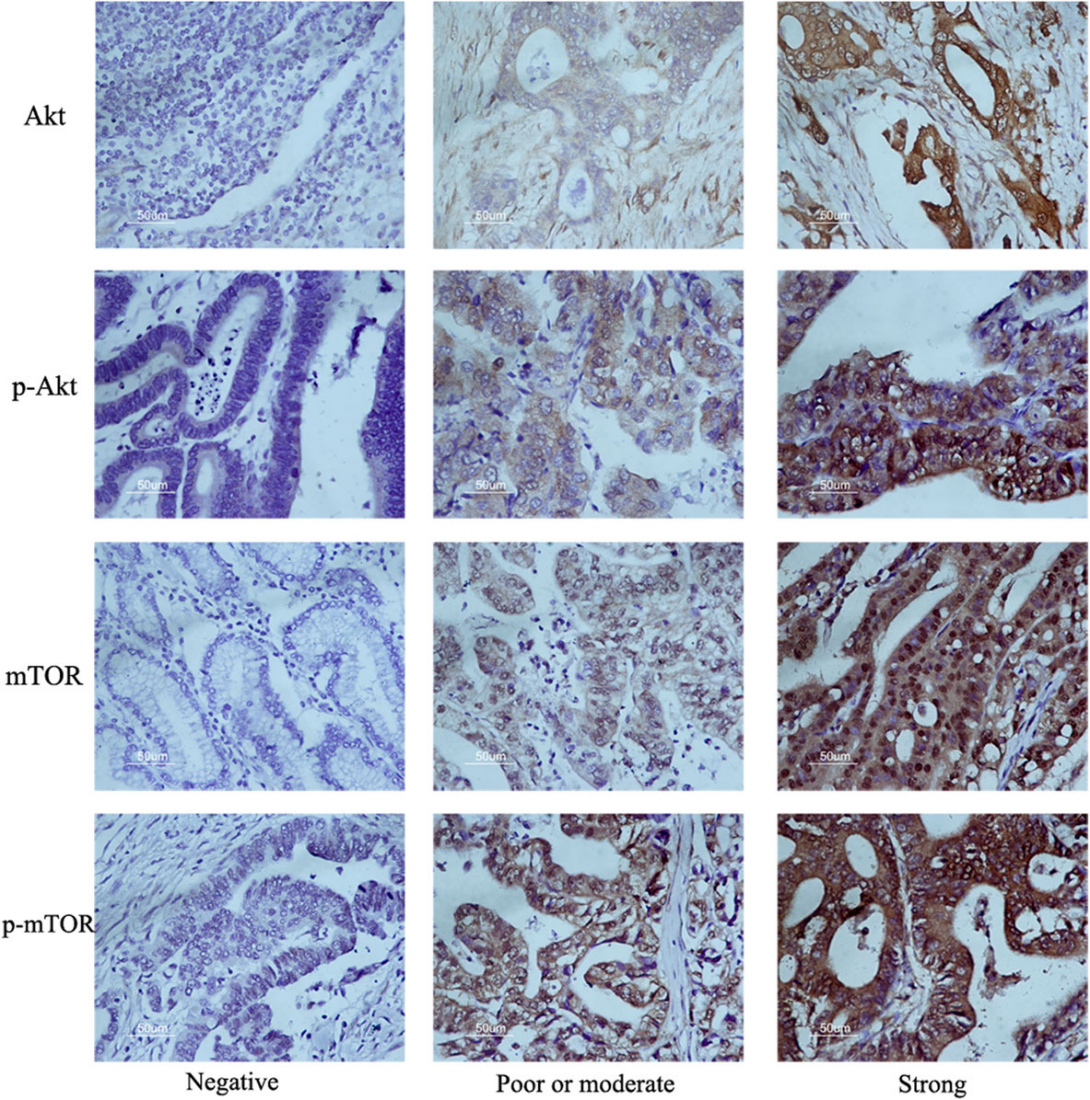
Immunohistochemical staining of PI3K/Akt/mTOR pathway related proteins in gastric cancer. Original magnification, ×200

The tissue samples were fixed in 10% neutral formalin and embedded in paraffin before further investigation. All tumor sections (thickness = 3–5 μm) were stained as directed in the manufacturer’s instructions. Tissue sections were deparaffinized and hydrated in xylene and serially diluted grades of ethanol, respectively. Endogenous peroxidase was blocked by incubation in 3% H_2_O_2_ for 10 min at room temperature. Antigen retrieval was performed in a microwave oven using citrate solution. Tissue sections were incubated with the appropriate antibody overnight at 4 °C. Next, slides were washed three times in phosphate-buffered saline (PBS), and then incubatedin secondary antibody for 20 min. After three further washes in PBS, a diaminobenzidine tetrahydrochloride (DAB) working solution was applied. Finally, the sections were counterstained with hematoxylin. The following primary antibodies were used: HER2 (Rabbit monoclonal antibody, 1:150 dilution, bs-0125R, Bioss), HER3 (Rabbit monoclonal antibody, 1:200 dilution, bs-1454R, Bioss), Akt (Rabbit monoclonal antibody, 1:100 dilution, Y89, Abcam), p-Akt (Rabbit monoclonal antibody, 1:150 dilution, EP2109, Abcam), mTOR (Rabbit monoclonal antibody, 1:200 dilution, Y391, Abcam), p-mTOR (Rabbit monoclonal antibody, 1:200 dilution, EPR426(2), Abcam). The results of IHC was performed in Fig. 1 and Fig. 2.

### Evaluation of immunohistochemistry

HER2 and HER3 levels were scored as follows: 0, no staining or in <10% of tumor cells; 1+, faint/barely perceptible partial staining in <10% of tumor cells; 2+, weak to moderate staining of the entire membrane or cytoplasm in >10% of tumor cells; 3+, strong staining in >10% of tumor cells. Moderate staining (2+) and strong staining (3+) were considered positive expression.

The results of immunohistochemical staining for mTOR and p-mTOR were evaluated by two independent investigators according to a semi-quantitative grading system based on both the proportion of stained cells and staining intensity [18]. Staining intensity was scored as 0 (negative), 1 (weak), 2 (moderate), or 3 (strong), and the percentage of positive epithelial cells as 0 (no staining), 1(<1/3 staining), 2 (1/3 to 2/3 staining), and 3 (>2/3 staining). A Histo score was generated as the product of staining intensity by percentage of positive epithelial cells. The samples after immunostaining were divided into two groups: score of 0–2, negative expression; score > 2, positive expression.

### Quantitative reverse transcription-PCR

Quantitative reverse transcription PCR (qRT-PCR) was performed as previously described on selected gastric cancer tissues [19]. Total RNA was extracted with TRIzol reagent (Invitrogen). Then, cDNA was obtained with PrimeScript RT-polymerase (Takara); qRT-PCR amplification was performed with SYBR Green Mix (Takara Bio, Dalian, China). The cycle-threshold (Ct) value for each gene was normalized to β-actin levels, and data analysis was carried out by the 2-ΔCt method. The primers used in qRT-PCR are shown in Table 1.

**Table 1:** Primers used in qRT-PCR

Gene	Forward primer (5′—3′)	Reverse primer (5′—3′)
GAPDH	GGTCACCAGGGCTGCTTTTA	TTCCCGTTCTCAGCCTTGAC
HER-2	CCGAGGGCCGGTATACATTC	GCTTGCTGCACTTCTCACAC
HER-3	CCCAGGTCTACGATGGGAAG	AGAAGGAACCATCGGGAACT
AKT	ACTGTCATCGAACGCACCTT	CTCCTCCTCCTCCTGCTTCT
mTOR	ACCCATCCAACCTGATGCTG	ACACTGTCCTTGTGCTCTCG

### Statistical analysis

Statistical analyses were performed with SPSS version 16.0 (SPSS Inc., Chicago, IL, USA). Associations of clinical variables and target proteins were examined by the Chi-square test (Pearson’s Chi-square analysis, Continuity correction or Fisher’s exact was used according to sample size and theoretical frequency). Spearman’s rank correlation analysis was used to assess the relationships among proteins. Survival curves were generated by the Kaplan–Meier method, with statistical significance evaluated by the log-rank test. Univariate analysis was based on a Cox proportional hazard regression model, and multivariate survival analysis was conducted by Cox regression analysis with the forward stepwise method. *P* < 0.05 was considered statistically significant.

### Meta-analysis

#### Aims

Because of the small number of patients, the correlation between HER2, HER3, HER2-HER3 co-expression, pathway related proteins and clinicopathological parameters are not credible or the difference was not significant. In addition, the evaluation of HER2 and HER3 is not very appropriate, because no FISH data provided. A meta-analysis was conducted to fully investigate whether HER3, HER2-HER3 co-expression, pathway related proteins have relationships with clinicopathological parameters and OS.

### Methods

Eligible studies were searched on PubMed, Ovid, Web of Science, and Cochrane databases through multiple search strategies. The search terms: (1) (“HER3” or “ErbB3” or “Human epidermal growth factor receptor”) and (“gastric” or “stomach” or “cardia” or “gastrointestinal”) and (“adenocarcinoma” or “carcinoma” or “cancer” or “tumour” or “neoplasm” or “tumor”); (2) (“HER” or “ErbB” or “Human epidermal growth factor receptor” or “HER family”) and (“gastric” or “stomach” or “cardia” or “gastrointestinal” or “colorectal” or “digestive tract”) and (“adenocarcinoma” or “carcinoma” or “cancer” or “tumour” or “neoplasm” or “tumor”); (3) (“Akt” OR “protein kinase B” OR “p-Akt” OR “phosphrylated Akt” OR “phosphrylated protein kinase B”)AND (“gastric” OR “stomach” OR “cardia”) AND (“adenocarcinoma” OR “carcinoma” OR “cancer” OR “tumour” OR “neoplasm” OR “tumor”); (4) (“mTOR” OR “the mammmalian target of Rapamycin” OR “p-mTOR” OR “phosphrylated mTOR” OR “phosphrylated mammmalian target of Rapamycin”) AND (“gastric” OR “stomach” OR “cardia”) AND (“adenocarcinoma” OR “carcinoma” OR “cancer” OR “tumour” OR “neoplasm” OR “tumor”). The full texts of the studies were read to find whether the studies met the inclusion criteria.

The full texts of the studies were read to find whether the studies met the following inclusion criteria: (1) GC/Digestive cancer was identified, (2) expression of proteins was evaluated by IHC, (3) information on clinicopathological parameters and OS was provided, (4) standards to assess the status of proteins was consistent in different studies, and (5) article was published in English and Chinese. The studies were excluded if they met the exclusion criteria: (1) repetition, (2) reviews, (3) case reports, and (4) evaluation method was not IHC.

Two investigators (Guo-dong Cao and Ke Chen) extracted the data independently after the disagreements were resolved. The following data were extracted: first author’s name, year of publication, total number of patients, clinicopathological parameters, and survival time. During the process of data extraction, disagreements were discussed with a third investigator (Mao-ming Xiong) until a consensus was reached. Two investigators assessed the quality of included studies using the Newcastle–Ottawa scale [20].

All the statistical analyses were performed using the STATA software (version 11.0, StataCorp LP, College Station, TX, USA). The crude odds ratio (OR) and 95% confidence interval (CI) were used to estimate the strength of the associations between HER3, Akt, p-Akt, mTOR, p-mTOR and clinicopathological parameters of GC patients. Risk ratios (RR) and 95% CIs were used in this meta-analysis to estimate the associations of the status of HER3, HER2-HER3 co-expression and pathway related proteins with OS. *I* ^2^ value, which indicated the percentage of total variation across studies, was used to assess statistical heterogeneity. Random-effects models (*I* ^2^ > 50% or *P* < 0.10) of analysis were used if significant heterogeneity was detected. Otherwise, fixed-effects models were used. In consideration of the potential publication bias, Begg’s rank correlation method and Egger’s weighted regression method were used (*P* < 0.05 indicates statistically significant publication bias).

## Results

### Expression levels of different target proteins in gastric cancer

**Fig. 3:**
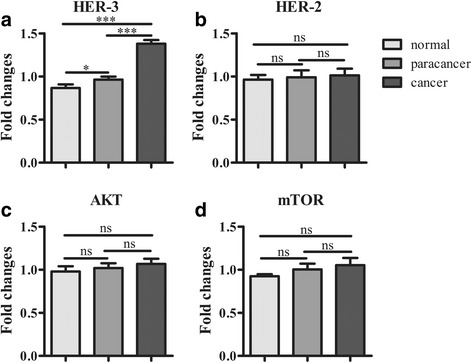
Expression of targets genes in the tissues. HER3 was highly expressed in the gastric cancer tissue (**a**). However, there were no significant differences among the three different tissues in HER2 (**b**), Akt (**c**) and mTOR (**d**) mRNA levels as assessed by qRT-PCR. *P < 0.05, **P < 0.01, ***P < 0.001

According to IHC results, HER2, HER3, Akt, p-Akt, mTOR and p-mTOR were differently expressed in GC tissue samples. The overall rates of HER2 and HER3 were 24.2% and 54.2%, respectively. IHC showed that the rates of Akt and p-Akt expression in GC were 66.7% and 59.2%, respectively. The overall rate of mTOR overexpression in 120 GC patients was 60.8%, and that of p-mTOR overexpression was 54.2%. No significant difference was found between this and previous studies. Furthermore, as shown in Fig. 3 based on qRT-PCR results, higher expression of HER3 was found in gastric cancer tissues than normal and para-carcinoma tissues. However, there were no overt differences among the three different tissues in HER2, Akt and mTOR mRNA levels as assessed by qRT-PCR.

### Associations of HER2 and HER3 levels with relevant parameters

Table 2 shows associations of HER2 overexpression with relevant parameters, and Table 3 summarizes Spearman’s correlation analysis between HER family members and PI3K/Akt/mTOR pathway-related proteins. HER2 overexpression was significantly related to differentiation (*P* = 0.01) and distant metastasis (*P* = 0.045), but not associated with the remaining clinicopathological variables, including depth of invasion (*P* = 0.47), lymph node metastasis (*P* = 0.98) and TNM stage (*P* = 0.16). However, positive HER2 expression was not only associated with HER3 overexpression (*P* < 0.001), but also overtly related to phosphorylated Akt and phosphorylated mTOR overexpression, but not Akt and mTOR (*P* = 0.036 vs. *P* = 0.765 and *P* < 0.001 vs. *P* = 0.877).

**Table 2:** Association between clinicopathological parameters, proteins and HER2, HER3, HER2-HER3 co-expression in 120 cases of gastric cancer

		Total patients	HER2-positive	HER2-negative	*P* value	HER3-positive	HER3-negative	*P* value	HER2/HER3 co-expression	*P* value
Sex	Male	80	19	61	0.88	42	38	0.60	16	0.75
	Female	40	10	30		23	17		9	
Age	<60y	49	9	40	0.02	24	25	0.34	7	0.14
	>60y	71	20	51		41	30		18	
Tumor size	<3 cm	15	4	11	1.00	7	8	0.53	4	0.80
	>3 cm	105	25	80		58	47		21	
Differentiation	Well/moderate	102	20	82	0.01	52	50	0.10	17	0.02
	Poor	18	9	9		13	5		8	
Tumor location	Upper/Medium	77	17	60	0.47	42	35	0.91	16	0.98
	Low	43	12	31		23	20		9	
Depth of invasion	T1 + T2	22	4	18	0.47	7	15	0.02	2	0.23
	T3 + T4	98	25	73		58	40		23	
Lymph node metastasis	N0	25	6	19	0.98	11	14	0.25	6	0.59
	N1 + N2 + N3	95	23	72		54	41		19	
Metastasis	M0	114	25	89	0.045	62	52	1.00	23	0.80
	M1	6	4	2		3	3		2	
TNM stage	I + II	33	5	28	0.16	17	16	0.72	5	0.35
	III + IV	87	24	63		48	39		20

**Table 3:** Spearman correlation analysis between HER family members and PI3K/Akt/mTOR pathway-related proteins

	HER2		HER3		HER2-HER3	
	Spearman correlation	*P* value	Spearman correlation	*P* value	Spearman correlation	*P* value
HER3	0.363	<0.001	NA	NA	NA	NA
Akt	0.028	0.765	0.201	0.028	0.044	0.632
p-Akt	0.192	0.036	0.359	<0.001	0.246	0.007
mTOR	0.014	0.877	0.187	0.041	0.017	0.853
p-mTOR	0.324	<0.001	0.362	<0.001	0.334	<0.001

Similar results were found for HER3 overexpression and HER2-HER3 co-expression. HER3 overexpression showed no association with clinicopathological parameters, except the depth of invasion (*P* = 0.02). However, HER3 expression was also associated with high phosphorylated Akt and phosphorylated mTOR levels, but not Akt and mTOR (*P* < 0.001 vs. *P* = 0.028 and *P* < 0.001 vs. *P* = 0.041).

In addition, HER2-HER3 co-expression showed significant associations with p-Akt and p-mTOR overexpression, but not Akt and mTOR (*P* = 0.007 vs.0.632, and *P* < 0.001 vs.0.853).

### Associations of PI3K/Akt/mTOR pathway-related proteins with clinicopathological parameters

As shown in Table 4, associations of pathway-related proteins, such as Akt, p-Akt, mTOR, p-mTOR and clinicopathological parameters were found. Akt and p-Akt had no associations with several vital clinical variables which are meaningful to prognosis, such as depth of invasion, lymph node metastasis, distant metastasis, and TNM stage.

**Table 4:** Association between clinicopathological parameters and Akt, p-Akt, mTOR, p-mTOR expression in 120 cases of gastric cancer

		Total patients	AKT-positive	AKT-negative	*P* value	p-AKT-positive	p-AKT-negative	*P* value	mTOR-positive	mTOR-negative	*P* value	p-mTOR-positive	p-mTOR-negative	*P* value
Sex	Male	80	55	25	0.49	46	34	0.60	50	30	0.60	46	34	0.30
	Female	40	25	15		25	15		23	17		19	21	
Age	<60y	49	25	24	<0.01	28	21	0.71	21	28	<0.01	26	23	0.84
	>60y	71	55	16		43	28		52	19		39	32	
Tumor size	<3 cm	15	11	4	1.00	9	6	0.94	9	6	0.58	9	6	0.63
	>3 cm	105	69	36		62	43		64	41		56	49	
Differentiation	Well/moderate	102	69	33	0.59	59	43	0.48	60	42	0.28	49	53	<0.01
	Poor	18	11	7		12	6		13	5		16	2	
Tumor location	Upper/Medium	77	46	31	0.03	46	31	0.86	49	28	0.40	43	34	0.62
	Low	43	34	9		25	18		24	19		22	21	
Depth of invasion	T1 + T2	22	14	8	0.74	9	13	0.054	10	12	0.10	6	16	<0.01
	T3 + T4	98	66	32		62	36		63	35		59	39	
LN metastasis	N0	25	18	7	0.53	12	13	0.20	12	13	0.14	9	16	0.04
	N1 + N2 + N3	95	62	33		59	36		61	34		56	39	
Metastasis	M0	114	77	37	0.66	70	44	0.08	70	44	0.58	63	51	0.53
	M1	6	3	3		1	5		3	3		2	4	
TNM stage	I + II	33	21	12	0.67	20	13	0.84	15	18	0.03	12	21	0.02
	III + IV	87	59	28		51	36		58	29		53	34	

However, high mTOR and p-mTOR expression levels exhibited significant associations with some clinicopathological parameters. Positive mTOR expression was tightly related to TNM stage (*P* = 0.001); positive mTOR expression always reflected later TNM stage, unlike no mTOR expression. Moreover, p-mTOR had tight associations with differentiation (*P* < 0.01), depth of invasion (*P* < 0.01), lymph node metastasis (*P* = 0.04) and TNM stage (*P* = 0.02); patients with positive p-mTOR expression may show poor differentiation, deeper wall invasion, positive lymph node metastasis, and late tumor stage.

### Survival analysis

**Fig. 4:**
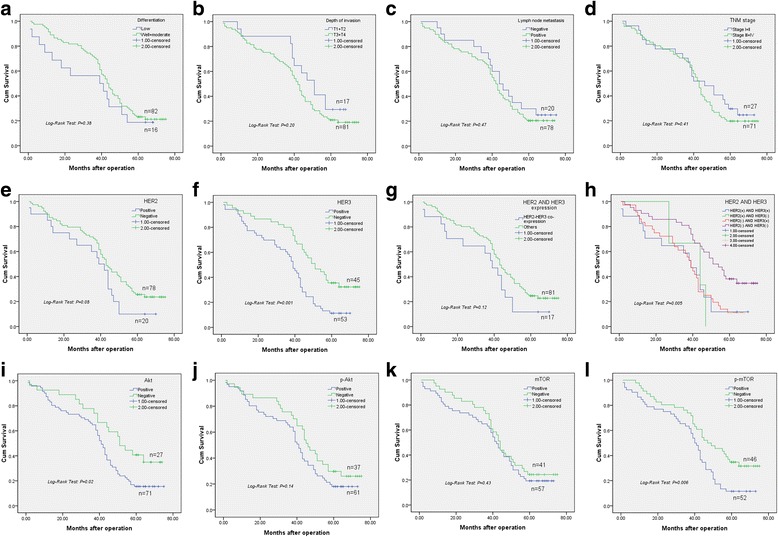
Kaplan-Meier survival curves for overall survival of GC patients. Following clinical parameters have no association with OS: differentiation (**a**), depth of invasion (**b**), lymph node metastasis (**c**) and TNM stage (**d**). However, overexpression of HER family members, such as HER3 (*P* = 0.001, **f**) and HER2-HER3 co-expression (*P* = 0.005, **h**) are significantly related to overall survival rate of GC patients. Akt (**i**), p-Akt (**j**) and mTOR (**k**) are not associated with OS. Meanwhile, p-mTOR (**l**) has tight link with overall survival (*P* = 0.006)

Survival analysis was performed to assess whether vital clinical parameters, HER family members, and PI3K/Akt/mTOR pathway related proteins are associated with patient outcomes (Fig. 4). In this analysis performed by the Log-rank test, differentiation (*P* = 0.38, Fig. 4a), depth of invasion (*P* = 0.20, Fig. 4b), lymph node metastasis (*P* = 0.47, Fig. 4c) and TNM stage (*P* = 0.41, Fig. 4d) had no relationships with OS in GC patients.

However, HER family members showed associations with overall survival. Positive HER2 expression had a tendency to decrease the survival time, although not significant (Log-rank test, *P* = 0.08, Fig. 4e). Patients with HER3 overexpression had obviously shorter survival compared with GC patients not expressing HER3 (Log-rank test, *P* = 0.001, Fig. 4f). HER2-HER3 co-expression also significantly shortened the survival time and overall survival rate compared with the remaining GC patients (Log-rank test, *P* = 0.12, Fig. 4g); significant differences were also found among detailed categories, including HER2-HER3 co-expression, HER2 positive and HER3 negative, HER2 negative and HER3 positive, and HER2 and HER3 negative (Log-rank test: *P* = 0.005, Fig. 4h).

In PI3K/Akt/mTOR pathway-related proteins, Akt expression had significant association with low survival rate (Log-rank test, *P* = 0.02, Fig. 4i). However, p-Akt (Log-rank test, *P* = 0.14, Fig. 4j) and mTOR (Log-rank test, *P* = 0.43, Fig. 4k) were not associated with OS in GC patients. Overt associations were found of OS with positive p-mTOR expression (Log-rank test, *P* = 0.006, Fig. 4l); its overexpression always led to reduced survival of GC patients.

### Univariate and multivariate analyses of OS in GC patients

The prognostic values of HER proteins, PI3K/Akt/mTOR pathway molecules and several clinical factors were evaluated by univariate and multivariate analyses, respectively. In univariate analysis, all the above factors showed no relationship with OS, except HER3 (Hazard ratio = 2.20, 95%CI 1.38–3.52, *P* = 0.001, Table 5) and p-mTOR (Hazard ratio = 1.88, 95%CI 1.19–2.99, *P* = 0.01, Table 5); high expression levels of these proteins were associated with OS. Multivariate Cox proportional hazard model also showed a potential connection between HER2, HER3, or HER2-HER3 co-expression and OS, although not statistically significant (*P* values were very close to 0.05).

**Table 5:** Univariate analysis and multivariate analysis of overall survival in 120 gastric cancer patients

Variables	Univariate analysis			Multivariate analysis		
	Hazard Ratio	95% CI	*P* value	Hazard Ratio	95% CI	*P* value
Sex	1.15	0.70–1.89	0.58	1.03	0.60–1.77	0.91
Age	0.93	0.59–1.46	0.75	0.76	0.45–1.29	0.31
Tumor size	0.94	0.47–1.90	0.87	0.77	0.35–1.71	0.52
Differentiation	1.30	0.72–2.37	0.38	1.07	0.54–2.14	0.84
Tumor location	1.09	0.68–1.75	0.71	1.10	0.54–2.14	0.84
Depth of invasion	1.49	0.80–2.76	0.21	1.81	0.76–4.33	0.18
LN metastasis	1.31	0.73–2.34	0.36	1.31	0.62–2.79	0.48
Distant metastasis	0.83	0.26–2.63	0.75	0.66	0.97–4.45	0.67
TNM stage	1.30	0.77–2.19	0.33	0.70	0.30–1.63	0.41
HER2 expression	1.61	0.94–2.74	0.08	5.98	0.97–36.72	0.054
HER3 expression	2.20	1.38–3.52	0.001	1.82	0.93–3.57	0.08
HER2-HER3 co-expression	1.56	0.88–2.75	0.13	6.21	0.95–40.89	0.057
Akt expression	1.79	1.04–3.08	0.04	1.64	0.87–3.12	0.13
p-Akt expression	1.42	0.87–2.27	0.15	0.88	0.46–1.68	0.69
mTOR expression	1.33	0.84–2.11	0.22	1.14	0.64–2.03	0.67
p-mTOR expression	1.88	1.19–2.99	0.01	1.47	0.75–2.87	0.26

### Characteristics of studies in meta-analyses

After reviewing the abstracts and full texts based on the set inclusion and exclusion criteria, 5 studies evaluating HER3 overexpression and GC [21–25], 4 detailing HER2-HER3 co-expression [14–16, 25], 3 describing mTOR [26–28], 7 researching p-mTOR [28–34], 2 exploring Akt [34, 35], and 12 that researched p-Akt overexpression in GC [33, 34, 36–45] were finally selected (Additional file 2: Figure S1). The characteristics of these eligible publications are shown in Additional file 3: Table S1-S5. Clinicopathological variables were extracted as follows: gender, age, tumor location, differentiation, depth of invasion, lymph node metastasis, distant metastasis, and TNM stage. In the included studies, the samples were analyzed by IHC, and the standards for assessing the status of expression were almost consistent. The overall rates of HER3, Akt, p-Akt, mTOR, p-mTOR positive expression in patients with GC were 26.7%, 67.2%, 54.8%, 53.9% and 48.3%, respectively. HER3 overexpression rate ranged from 11.7% to 62%; the positive rates of Akt ranged from 37.1% to 75.4%, and those of p-Akt from 28.9% to 88.1%. Meanwhile, mTOR expression ranged from 50.8% to 73.6% of GC patients, and p-mTOR from 45.2% to 76.4%.

### Associations of HER3 and PI3K/Akt/mTOR pathway-related proteins with the clinicopathological parameters analyzed by meta-analysis

**Fig. 5:**
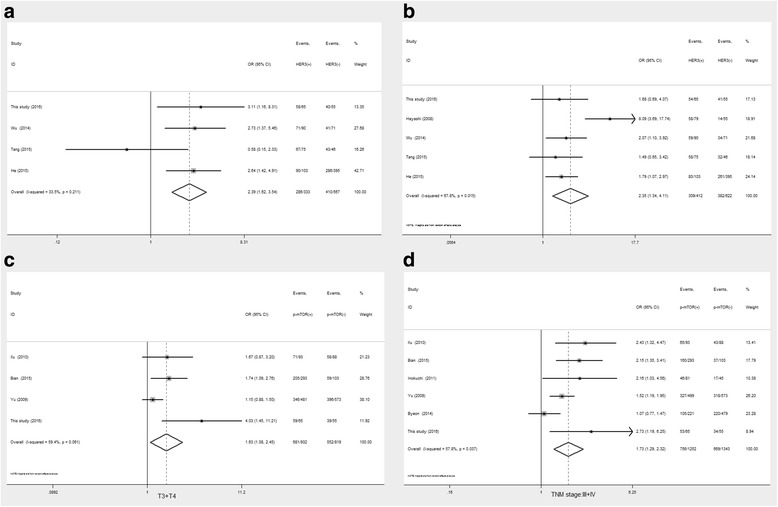
Forrest plot of odds ratio for the association of target proteins and clinicopathological variables Association between HER3overexpression and depth of invasion (**a**) and lymph node metastasis (**b**), association of p-mTOR overexpression and depth of invasion (**c**) and TNM stage (**d**)

As shown in Table 6, positive HER3 expression was related to depth of invasion (OR = 2.39, 95%CI 1.62–3.54, *P* < 0.001, Fig. 5a) and lymph node metastasis (OR = 2.35, 95%CI 1.34–4.11, *P* = 0.003, Fig. 5b). However, no significant associations were found of Akt overexpression with clinicopathological variables. In addition, no significant relationships were obtained between p-Akt overexpression and clinicopathological variables.

**Table 6:** Meta-analysis of association between clinicopathological parameters and HER3, Akt, p-Akt, mTOR, p-mTOR expression in gastric cancer

Target proteins	Parameters	Number of studies	Number of patients	Heterogeneity		Model	OR(95%CI)	*P* value
				*I* ^2^ (%)	*P* value			
HER3	Sex (male/female)	5	1034	18	0.30	FE	0.89(0.66,1.20)	0.44
	Depth of invasion (T3 + T4/T1 + T2)	4	900	34	0.20	FE	2.39(1.62,3.54)	<0.001
	LN metastasis(positive/negative)	5	1034	68	0.02	RE	2.35(1.34,4.11)	0.003
	Metastasis (positive/negative)	5	1034	47	0.10	FE	1.39(0.66,2.91)	0.39
	Tumor stage (III + IV/I + II)	4	801	54	0.09	RE	1.38(0.76,2.49)	0.29
Akt	Sex (male/female)	3	501	0	0.67	FE	0.99(0.66,1.49)	0.96
	Age (>60/<60)	2	190	89	0	RE	1.24(0.17,8.88)	0.83
	Tumor location (upper/low)	2	431	71	0.05	RE	0.69(0.26,1.79)	0.44
	Differentiation (poor/well)	2	190	77	0.04	RE	1.63(0.35,7.53)	0.53
	Depth of invasion (T3 + T4/T1 + T2)	1	120	–	–	–	1.18(0.45,3.10)	0.74
	LN metastasis(positive/negative)	3	501	68	0.05	RE	1.27(0.44,3.61)	0.66
	Metastasis (positive/negative)	3	501	72	0.03	RE	1.04(0.24,4.46)	0.96
	Tumor stage (III + IV/I + II)	3	501	70	0.04	RE	1.17(0.45,3.00)	0.75
p-Akt	Sex (male/female)	9	1477	0	0.79	FE	1.29(1.03,1.63)	0.03
	Age (>60/<60)	5	1326	0	0.79	FE	0.95(0.74,1.20)	0.65
	Tumor location (upper/low)	4	895	0	0.70	FE	1.03(0.78,1.37)	0.81
	Differentiation (poor/well)	7	996	65	0.01	RE	1.14(0.65,2.02)	0.64
	Depth of invasion (T3 + T4/T1 + T2)	7	1283	60	0.02	RE	1.23(0.80,1.90)	0.35
	LN metastasis(positive/negative)	12	2019	69	0.00	RE	1.29(0.88,1.89)	0.19
	Metastasis (positive/negative)	5	1351	66	0.02	RE	0.75(0.29,1.89)	0.54
	Tumor stage (III + IV/I + II)	11	1597	62	0.003	RE	1.20(0.81,1.79)	0.36
mTOR	Sex (male/female)	4	1637	0	0.95	FE	1.16(0.94,1.43)	0.17
	Age (>60/<60)	2	1192	88	0.004	RE	1.90(0.59,6.06)	0.28
	Tumor location (upper/low)	2	1148	0	0.87	FE	1.30(1.03,1.64)	0.03
	Differentiation (poor/well)	3	1225	88	0	RE	1.59(0.33,7.57)	0.56
	Depth of invasion (T3 + T4/T1 + T2)	3	1604	57	0.10	RE	0.88(0.59,1.31)	0.54
	LN metastasis(positive/negative)	4	1637	74	0.01	RE	1.72(0.98,3.01)	0.06
	Tumor stage (III + IV/I + II)	3	1225	89	0	RE	3.13(0.72,13.61)	0.13
p-mTOR	Sex (male/female)	8	2994	0	0.60	FE	1.09(0.93,1.28)	0.30
	Age (>60/<60)	5	2469	0	0.44	FE	1.46(1.24,1.72)	<0.001
	Tumor location (upper/low)	5	2001	11	0.35	FE	1.26(1.03,1.55)	0.03
	Differentiation (poor/well)	4	1663	75	0.01	RE	0.99(0.57,1.72)	0.87
	Depth of invasion (T3 + T4/T1 + T2)	4	1751	54	0.06	RE	1.63(1.08,2.45)	0.02
	LN metastasis(positive/negative)	7	2294	90	0	RE	1.57(0.83,2.98)	0.17
	Metastasis (positive/negative)	2	246	58	0.12	RE	1.05(0.25,4.44)	0.94
	Tumor stage (III + IV/I + II)	6	2595	58	0.04	RE	1.73(1.29,2.32)	<0.001

*OR* odds ratio, *CI* confidence interval, *FE* fixed-effect model, *RE* random-effect model, *LN* metastasis: lymph node metastasis

As shown in Table 6, mTOR showed a trend of association with lymph node metastasis (OR = 1.72, 95%CI 0.98–3.01, *P* = 0.06) and late TNM stage (OR = 3.13, 95%CI 0.72–13.61, *P* = 0.13). Meanwhile, p-mTOR was significantly associated with age (OR = 1.46, 95%CI 1.24–1.72, *P* < 0.001), tumor location (OR = 1.26, 95%CI 1.03–1.55, *P* = 0.03), depth of invasion (OR = 1.63, 95%CI 1.08–2.45, *P* = 0.02, Fig. 5c), and TNM stage (OR = 1.73, 95%CI 1.29–2.32, *P* < 0.001, Fig. 5d). It should be noted that p-mTOR overexpression showed a trend of increasing lymph node metastasis (OR = 1.57, 95%CI 0.83–2.98, *P* = 0.17). In addition, HER family members, for example HER3, had partly consistency with p-mTOR for the associations with clinicopathological parameters.

### Associations of HER family members and PI3K/Akt/mTOR pathway-related proteins with OS

**Fig. 6:**
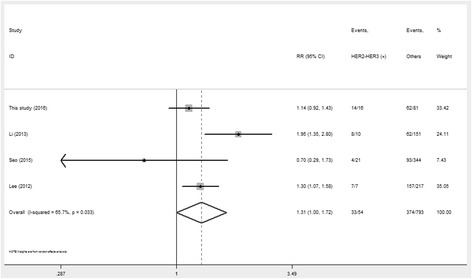
Forrest plot of the risk ratio for the association between HER2-HER3 co-expression and 5-year OS

**Fig. 7:**
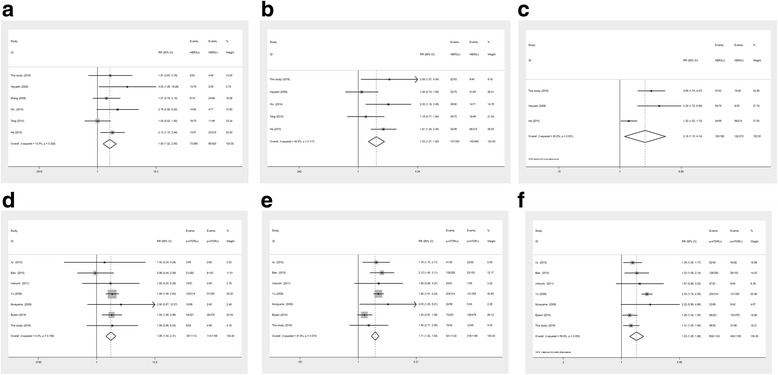
Forrest plot of the risk ratio for the association of HER3 and p-mTOR overexpression and OS: (**a**) Association between HER3 over-expression and 1-year OS (**b**) Association between HER3 over-expression and 3-year OS (**c**) Association between HER3 over-expression and 5-year OS (**d**) Association between p-mTOR over-expression and 1-year OS (**e**) Association between p-mTOR over-expression and 3-year OS (**f**) Association between p-mTOR over-expression and 5-year OS

Survival times were extracted from Kaplan–Meier survival curves analyzed with the Engage Digitizer software. As shown in Table 7, HER2-HER3 co-expression showed a gradually but obviously reduced OS rate, especially 5-year OS (OR = 1.31, 95%CI 1.00–1.72, *P* < 0.05, Fig. 6). GC patients with positive HER3 expression had apparently decreased 1-year (OR = 1.85, 95%CI 1.32–2.58, *P* < 0.001, Fig. 7a), 3-year (OR = 1.53, 95%CI 1.27–1.85, *P* < 0.001, Fig. 7b) and 5-year (OR = 2.18, 95%CI 1.15–4.14, *P* = 0.02, Fig. 7c) survival rates compared with patients negative for HER3.

**Table 7:** Meta-analysis of association between HER family members, pathway-related proteins expression and OS

Proteins	Tumor location	OS	Number of studies	Number of patients	Heterogeneity		Model	RR(95%CI)	*P* value
					*I* ^2^ (%)	*P* value			
HER2-HER3	Digestive tract	1-year OS	5	969	48	0.11	FE	0.71(0.36,1.40)	0.32
		3-year OS	5	969	0	0.48	RE	1.27(0.98,1.64)	0.07
		5-year OS	4	847	66	0.03	RE	1.31(1.00,1.72)	0.049
HER3	Stomach	1-year OS	6	888	14	0.33	FE	1.85(1.32,2.58)	<0.001
		3-year OS	5	813	46	0.12	FE	1.53(1.27,1.85)	<0.001
		5-year OS	3	503	85	0.001	RE	2.18(1.15,4.14)	0.02
Akt	Stomach	1-year OS	1	98	–	–	–	2.09(0.50,8.83)	0.32
		3-year OS	1	98	–	–	–	1.59(0.73,3.43)	0.24
		5-year OS	1	98	–	–	–	1.43(1.06,1.03)	0.02
p-Akt	Stomach	1-year OS	8	1558	66	0.004	RE	0.96(0.56,1.65)	0.89
		3-year OS	8	1558	75	0	RE	1.19(0.84,1.69)	0.32
		5-year OS	7	1513	79	0	RE	1.15(0.84,1.58)	0.37
mTOR	Stomach	1-year OS	3	1179	90	0	RE	1.02(0.38,1.2.73)	0.97
		3-year OS	3	1179	89	0	RE	1.06(0.62,1.81)	0.82
		5-year OS	3	1179	94	0	RE	1.02(0.65,1.61)	0.94
p-mTOR	Stomach	1-year OS	7	2269	0	0.75	FE	1.86(1.50,2.31)	<0.001
		3-year OS	7	2269	48	0.07	FE	1.71(1.52,1.93)	<0.001
		5-year OS	7	2269	70	0.003	RE	1.53(1.26,1.86)	<0.001

*OS* overall survival, *RR* risk ratio, *CI* confidence interval, *FE* fixed-effect model, *RE* random-effect model

Furthermore, Akt, p-Akt and mTOR had no obvious differences between the positive and negative expression groups, and their overexpression was not associated with OS in GC patients. It should also be noted that p-mTOR had an overtly reduced 1-year OS rate (RR = 1.86, 95%CI 1.50–2.31, *P* < 0.001, Fig. 7d); moreover, significant associations were found of p-mTOR overexpression with 3-year (RR = 1.71, 95%CI 1.52–1.93, *P* < 0.001, Fig. 7e) and 5-year (RR = 1.53, 95%CI: 1.26–1.86, *P* < 0.001, Fig. 7f) OS in GC patients.

### Sensitivity and publication bias analyses

**Fig. 8:**
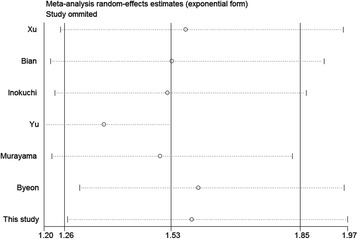
Effect of individual studies on pooled risk ratios (RR) for p-mTOR expression and overall survival (OS) in patients with gastric cancer

In order to assess the robustness of the RR estimates for OS, sensitivity analysis was conducted by individually excluding articles and analyzing the effects on the remaining studies. As shown in Fig. 8, sensitivity analysis indicated that the RR estimates were relatively reliable and credible, with no point estimates of the omitted individual studies laying outside the 95%CI.

**Fig. 9:**
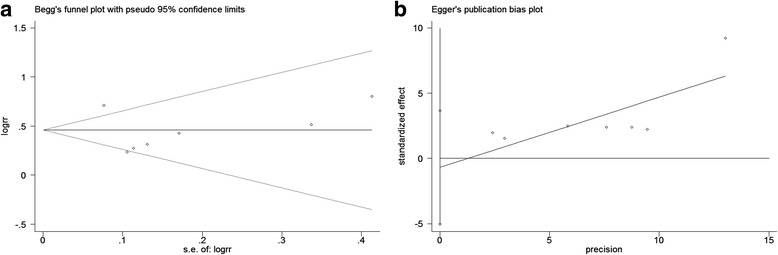
Begg’s funnel plot (*P* = 0.23, **a**) and Egger’s funnel plot (*P* = 0.70, **b**) for possible publication bias test of this study. There was no publication bias and the results are credible

The Begg’s rank correlation and Egger’s weighted regression methods were used to statistically assess publication bias. As shown in Fig. 9a and b, there was no publication bias in the current meta-analysis, indicating that the present results were credible.

## Discussion

Overexpression of HER family members is associated with the activation of several downstream pathways that lead to cell transformation and proliferation, which are associated with tumorigenesis [46]. Overexpression of HER protein family members in GC has been reported in multiple previous studies, although the conclusions remain controversial. A few studies paid attention to HER2-HER3 co-expression in tumors, and reported that formation of the HER2–HER3 heterodimer is associated with obviously decreased survival in breast cancer patients; moreover, preventing the dimerization shows clinical benefits [13]. Li et al. [14] and Lee et al. [15] obtained similar results in GC and EHCC, respectively. HER2-HER3 co-expression markedly reduces the survival rate of cancer patients. However, in a study of GC by Tang et al. [25], somewhat contradictory observations were made. The clinical significance of such overexpression thus remains unclear.

Previous researchers [6] have provided evidence for the presence of activating HER3 mutations, which promote tumorigenesis by inducing signaling pathways through HER2-HER3 heterodimer formation in a ligand-independent manner. HER3 is significantly correlated with HER2 overexpression or HER2 gene amplification in CRC [16]. In this study, a similar conclusion was reached by IHC, with HER2 overexpression significantly correlated with HER3 overexpression (*P* = 0.02). HER2 overexpression is usually associated with HER3 overexpression (25/29 vs. 44/92); this does not necessarily imply that HER2 and HER3 dimerize to form a heterodimer, and HER2 or HER3 homodimers might formally exist, although such homodimers are non-functional.

It is known that HER3 is a preferred dimerization partner that can sustain the activation of PI3K/Akt signaling [47, 48]. The intrinsic tyrosine kinase domain of HER3 is defective, and HER2 has no ligand. Moreover, HER3 usually heterodimerizes with HER2, and the HER2/HER3 heterodimer is likely the most effective complex among all heterodimers [49]. Moreover, the HER2/HER3 heterodimer is the most effective dimer sustaining the activation of PI3K/Akt signaling. However, the involved activation mechanism of this pathway and functional molecules remain unknown. Wu et al. [22] found that the phosphorylation levels of the Ser473 and Thr308 residues of Akt are decreased after Her3 knockdown. In addition, immunoblotting provided evidence that HER3 may be associated to the phosphorylation of pathway-related proteins. Few studies have investigated the correlation between mTOR or p-mTOR overexpression and GC. The mTOR protein is a down-stream effector of the PI3K-Akt signaling pathway and considered a Ser/Thr protein kinase; it has received considerable attention as a possible target for cancer treatment [50]. A phase III trial revealed that treatment with an mTOR inhibitor (i.e., everolimus) prolongs progression-free survival in patients with metastatic renal cell carcinoma [51].Yu et al. [28] reported that p-mTOR overexpression is related to clinicopathological variables, and p-mTOR appears to be a more sensitive biomarker than total mTOR in predicting the OS of patients. Overexpression of p-mTOR, but not mTOR, can be considered an independent prognostic factor in patients with GC, corroborating other findings that p-mTOR might formally serve as a potential prognostic predictor [29, 31, 34].

In the present study, we found that HER2, HER3 and HER2-HER3 co-expression were significantly associated with phosphorylated pathway-related proteins such as p-Akt and p-mTOR (all *P* < 0.05), in comparison with HER2, HER3 or HER2-HER3 negative expression groups. Meanwhile, HER2, HER3 and HER2-HER3 co-expression was not overtly associated with the overexpression of Akt or mTOR (Table 3). Our results are partly similar to previously reported studies demonstrating that HER2, HER3 and HER2-HER3 co-expression enhance the phosphorylation of Akt and mTOR. IHC data (Fig. 2 and Table 4) showed that Akt, p-Akt, and mTOR were not significantly associated with clinicopathological parameters. However, p-mTOR overexpression was clinically significant; indeed, GC patients with p-mTOR overexpression poorer differentiation, higher possibility of lymph node metastasis, deeper wall invasion and later tumor stage compared with p-mTOR negative patients. Besides survival analyses, univariate and multivariate analyses indicated that expression of HER family members (such as HER2, HER3 and HER2-HER3 co-expression state) as well as p-mTOR significantly decreased the survival of GC patients.

Due to the limitation of a small sample size, in order to comprehensively assess the prognostic values of pathway-related proteins, a meta-analysis was conducted. Interestingly, HER3 expression was tightly associated with depth of invasion and lymph node metastasis (*P* < 0.05). Meanwhile, p-mTOR was significantly associated with age, tumor location, depth of invasion, and TNM stage (all P < 0.05), with a trend of higher risk of lymph node metastasis. Higher TNM stage always reflects shorter OS. Significant associations were obtained of p-mTOR and HER3 overexpression with 1-, 3- and 5-year OS (all *P* < 0.05). Meanwhile, HER2-HER3 co-expression showed a gradually reduced OS (statistical significance was obtained for the 5-year OS). In summary, interlinks may exist between HER family members and p-mTOR, after comparing the consistency of clinical and prognostic significance.

**Fig. 10:**
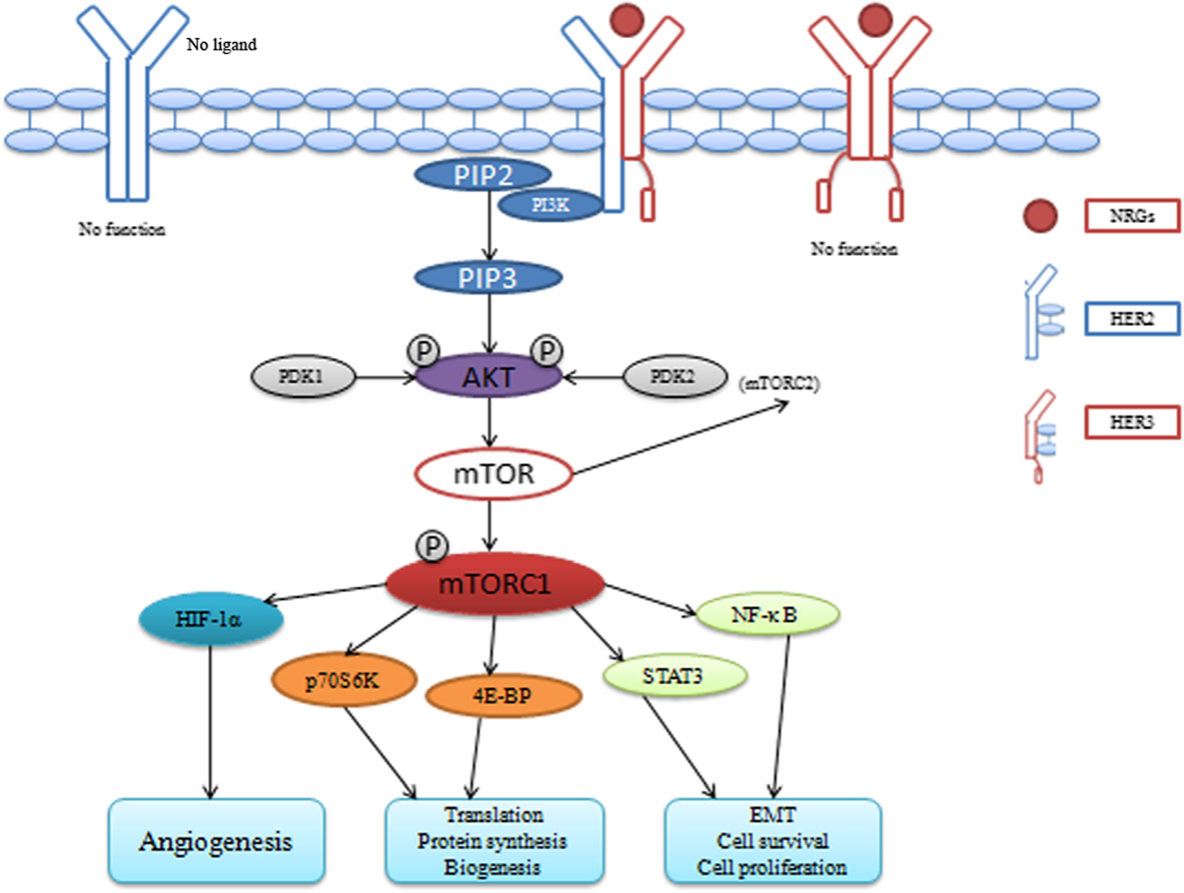
The function mechanism of HER2-HER3 co-expression. HER2-HER3 heterodimer is the effective dimer that can enhance the phosphorylation of Akt and mTOR, promotes translation, angiogenesis, EMT, cell proliferation. However, HER2 or HER3 hemodimier has no function that could not activiate PI3K/Akt/mTOR pathway, because of different deficiencies of themselves

As shown in Fig. 10, HER2-HER3 co-expression should be considered an independent oncological unit, due to the following points. First, after binding ligands, HER family members are activated by forming homo- or hetero-dimerization products, resulting in the phosphorylation of various downstream effectors, such as neuregulins (NRGs). NRG1 and NRG2 are specific ligands of HER3 [52, 53]. Secondly, when NRGs bind the extracellular receptor segment of HER3, some factors are phosphorylated and released, entering the nucleus and promoting cell growth, e.g. p48-ERBB3 binding protein 1 (p48-Ebp1), one of the distinct functional isoforms of EBP1 [54, 55]. Thirdly, the HER2/HER3 heterodimer is likely the most effective heterodimer – if positive HER2 expression exists alone without the overexpression of other HER family members, especially HER3; additionally, HER2 might just play a limited functional role. Finally, but most importantly, HER3 contains six consensus tyrosine phosphorylation sites, which bind the SH2 domain of the three regulatory subunits of PI3K [56, 57]. Binding of HER3 can activate the PI3K/Akt signaling pathway. Moreover, it should be acknowledged that HER2-mediated transformation of epithelial cells is associated with PI3K/Akt signaling induction in breast cancer. HER2 activation is not exclusively regulated by tyrosine phosphorylation, but also results from dimerization and binding. HER3 functions as the donor kinase and HER2 as the acceptor, in an asymmetric configuration [58].

Previous studies have proposed an alternative opinion that HER3 may function as an allosteric activator of other members of the HER receptor tyrosine kinase family [59–61]. HER3 contributes synergistically to HER2 and activates the PI3K/Akt pathway. In the present work, we obtained a conclusion partly similar to findings by previous studies of other solid tumors, such as breast cancer [13, 62].

Several advantages of this study should be acknowledged. (1) We provided evidence that HER2-HER3 co-expression could enhance the phosphorylation of Akt and mTOR, and confirmed that their co-expression might reduce OS in GC through p-mTOR overexpression, by IHC and a comprehensive meta-analysis. (2) This is the first available study and meta-analysis assessing the associations of HER3, Akt, p-Akt, mTOR, p-mTOR overexpression, and clinicopathological parameters in GC. (3) We compared the prognostic values of HER3 and p-mTOR in GC for the first time. However, the current study also had some limitations: (1) a small sample size; (2) no FISH data provided; (3) study method limited without any evidence supported by in vivo or in vitro investigations. Therefore, more articles and studies are required to confirm our findings.

## Conclusion

In summary, the findings reported in this study provided some compelling evidences that HER3 overexpression might be closely associated with HER2 status. Meanwhile, HER2-HER3 co-expression and p-mTOR overexpression were both associated with the prognosis of GC patients. According to previous studies and our data, we speculate that HER2-HER3 co-expression enhances the phosphorylation of Akt and mTOR, decreasing OS in GC patients through a p-mTOR-dependent signaling pathway.

## Additional files

Additional file 1: Certification of Ethics. (DOCX 219 kb)

Additional file 2: Flow diagrams of study selection procedure. (TIFF 590 kb)

Additional file 3: Table S1-S5. Clinicopathological parameters and quality scores of studies of each target protein in patients with gastric cancer. (DOCX 39 kb)

## References

[1] Strong VE, D’Amico TA, Kleinberg L, Ajani J (2013). Impact of the 7th edition AJCC staging classification on the NCCN clinical practice guidelines in oncology for gastric and esophageal cancers. J Natl Compr Cancer Netw.

[2] Liu TS, Wang Y, Chen SY, Sun YH (2008). An updated meta-analysis of adjuvant chemotherapy after curative resection for gastric cancer. Eur J Surg Oncol.

[3] Zhang XF, Huang CM, Lu HS, Wu XY, Wang C, Guang GX (2004). Surgical treatment and prognosis of gastric cancer in 2,613 patients. World J Gastroenterol.

[4] Kamangar F, Dores GM, Anderson WF (2006). Patterns of cancer incidence, mortality, and prevalence across five continents: defining priorities to reduce cancer disparities in different geographic regions of the world. J Clin Oncol.

[5] Lieto E, Ferraraccio F, Orditura M, Castellano P, Mura AL, Pinto M (2008). Expression of vascular endothelial growth factor (VEGF) and epidermal growth factor receptor (EGFR) is an independent prognostic indicator of worse outcome in gastric cancer patients. Ann Surg Oncol.

[6] Jaiswal BS, Kljavin NM, Stawiski EW, Chan E, Parikh C, Durinck S (2013). Oncogenic ERBB3 mutations in human cancers. Cancer Cell.

[7] Hynes NE, Lane HA (2005). ERBB receptors and cancer: the complexity of targeted inhibitors. Nat Rev Cancer.

[8] Montemurro F, Scaltriti M (2014). Biomarkers of drugs targeting HER-family signalling in cancer. J Pathol.

[9] Citri A, Skaria KB, Yarden Y (2003). The deaf and the dumb: the biology of ErbB-2 and ErbB-3. Exp Cell Res.

[10] Begnami MD, Fukuda E, Fregnani JH, Nonogaki S, Montagnini AL, da Costa WL (2011). Prognostic implications of altered human epidermal growth factor receptors (HERs) in gastric carcinomas: HER2 and HER3 are predictors of poor outcome. J Clin Oncol.

[11] Sithanandam G, Anderson LM (2008). The ERBB3 receptor in cancer and cancer gene therapy. Cancer Gene Ther.

[12] Amin DN, Campbell MR, Moasser MM (2010). The role of HER3, the unpretentious member of the HER family, in cancer biology and cancer therapeutics. Semin Cell Dev Biol.

[13] Baselga J, Swain SM (2009). Novel anticancer targets: revisiting ERBB2 and discovering ERBB3. Nat Rev Cancer.

[14] Li G, Gu RM, Wen X, Ming XZ, Xia L, Xu XY (2013). Clinical significance of human epidermal growth factor receptor family molecules expression in gastric cancer. Zhonghua Wei Chang Wai Ke Za Zhi.

[15] Lee HJ, Chung JY, Hewitt SM, Yu E, Hong SM (2012). HER3 overexpression is a prognostic indicator of extrahepatic cholangiocarcinoma. Virchows Arch.

[16] Seo AN, Kwak Y, Kim WH, Kim DW, Kang SB, Choe G (2015). HER3 protein expression in relation to HER2 positivity in patients with primary colorectal cancer: clinical relevance and prognostic value. Virchows Arch.

[17] Ahn HS, Lee HJ, Hahn S, Kim WH, Lee KU, Sano T (2010). Evaluation of the seventh American joint committee on cancer/International Union against Cancer classification of gastric adenocarcinoma in comparison with the sixth classification. Cancer.

[18] Gulhati P, Cai Q, Li J, Liu J, Rychahou PG, Qiu S, Gao T, Evers BM (2009). Targeted inhibition of mammalian target of rapamycin signaling inhibits tumorigenesis of colorectal cancer. Clin Cancer Res.

[19] Fang KP, Dai W, Ren YH, Xu YC, Zhang SM, Qian YB (2016). Both Talin-1 and Talin-2 correlate with malignancy potential of the human hepatocellular carcinoma MHCC-97 L cell. BMC Cancer.

[20] Stang A (2010). Critical evaluation of the Newcastle-Ottawa scale for the assessment of the quality of nonrandomized studies in meta-analyses. Eur J Epidemiol.

[21] Zhang XL, Yang YS, Xu DP, Qu JH, Guo MZ, Gong Y (2009). Comparative study on overexpression of HER2/neu and HER3 in gastric cancer. World J Surg.

[22] Wu X, Chen Y, Li G, Xia L, Gu R, Wen X (2014). Her3 is associated with poor survival of gastric adenocarcinoma: Her3 promotes proliferation, survival and migration of human gastric cancer mediated by PI3K/AKT signaling pathway. Med Oncol.

[23] Hayashi M, Inokuchi M, Takagi Y, Yamada H, Kojima K, Kumagai J (2008). High expression of HER3 is associated with a decreased survival in gastric cancer. Clin Cancer Res.

[24] He XX, Ding L, Lin Y, Shu M, Wen JM, Xue L (2015). Protein expression of HER2, 3, 4 in gastric cancer: correlation with clinical features and survival. J Clin Pathol.

[25] Tang D, Liu CY, Shen D, Fan S, Su X, Ye P (2015). Assessment and prognostic analysis of EGFR, HER2, and HER3 protein expression in surgically resected gastric adenocarcinomas. Onco Targets Ther.

[26] Li M, Sun H, Song L, Gao X, Chang W, Qin X (2012). Immunohistochemical expression of mTOR negatively correlates with PTEN expression in gastric carcinoma. Oncol Lett.

[27] Xiao L, Wang YC, Li WS, Du Y (2009). The role of mTOR and phospho-p70S6K in pathogenesis and progression of gastric carcinomas: an immunohistochemical study on tissue microarray. J Exp Clin Cancer Res.

[28] Yu G, Wang J, Chen Y, Wang X, Pan J, Li G (2009). Overexpression of phosphorylated mammalian target of rapamycin predicts lymph node metastasis and prognosis of chinese patients with gastric cancer. Clin Cancer Res.

[29] Xu DZ, Geng QR, Tian Y, Cai MY, Fang XJ, Zhan Q (2010). Activated mammalian target of rapamycin is a potential therapeutic target in gastric cancer. BMC Cancer.

[30] Inokuchi M, Murayama T, Hayashi M, Takagi Y, Kato K, Enjoj M (2011). Prognostic value of co-expression of STAT3, mTOR and EGFR in gastric cancer. Exp Ther Med.

[31] An JY, Kim KM, Choi MG, Noh JH, Sohn TS, Bae JM (2010). Prognostic role of p-mTOR expression in cancer tissues and metastatic lymph nodes in pT2b gastric cancer. Int J Cancer.

[32] Byeon SJ, Han N, Choi J, Kim MA, Kim WH (2014). Prognostic implication of TSC1 and mTOR expression in gastric carcinoma. J Surg Oncol.

[33] Bian Y, Wang Z, Xu J, Zhao W, Cao H (2015). Elevated Rictor expression is associated with tumor progression and poor prognosis in patients with gastric cancer. Biochem Biophys Res Commun.

[34] Murayama T, Inokuchi M, Takagi Y, Yamada H, Kojima K, Kumagai J (2009). Relation between outcomes and localisation of p-mTOR expression in gastric cancer. Br J Cancer.

[35] Gu Y, Jin S, Wang F, Hua Y, Yang L, Shu Y (2014). Clinicopathological significance of PI3K, Akt and survivin expression in gastric cancer. Biomed Pharmacother.

[36] Nam SY, Lee HS, Jung GA, Choi J, Cho SJ, Kim MK (2003). Akt/PKB activation in gastric carcinomas correlates with clinicopathologic variables and prognosis. APMIS.

[37] Sasaki T, Kuniyasu H, Luo Y, Kitayoshi M, Tanabe E, Kato D (2014). AKT activation and telomerase reverse transcriptase expression are concurrently associated with prognosis of gastric cancer. Pathobiology.

[38] Oki E, Baba H, Tokunaga E, Nakamura T, Ueda N, Futatsugi M (2005). Akt phosphorylation associates with LOH of PTEN and leads to chemoresistance for gastric cancer. Int J Cancer.

[39] Han Z, Wu K, Shen H, Li C, Han S, Hong L (2008). Akt1/protein kinase B alpha is involved in gastric cancer progression and cell proliferation. Dig Dis Sci.

[40] Hisamatsu Y, Oki E, Otsu H, Ando K, Saeki H, Tokunaga E (2016). Effect of EGFR and p-AKT Overexpression on chromosomal instability in gastric cancer. Ann Surg Oncol.

[41] Murakami D, Tsujitani S, Osaki T, Saito H, Katano K, Tatebe S (2007). Expression of phosphorylated Akt (pAkt) in gastric carcinoma predicts prognosis and efficacy of chemotherapy. Gastric Cancer.

[42] Chang H, Jung WY, Kang Y, Lee H, Kim A, Kim BH (2015). Expression of ROR1, pAkt, and pCREB in gastric adenocarcinoma. Ann Diagn Pathol.

[43] Sangawa A, Shintani M, Yamao N, Kamoshida S (2014). Phosphorylation status of Akt and caspase-9 in gastric and colorectal carcinomas. Int J Clin Exp Pathol.

[44] Zhou XD, Chen HX, Guan RN, Lei YP, Shu X, Zhu Y (2012). Protein kinase B phosphorylation correlates with vascular endothelial growth factor a and microvessel density in gastric adenocarcinoma. J Int Med Res.

[45] Kobayashi I, Semba S, Matsuda Y, Kuroda Y, Yokozaki H (2006). Significance of Akt phosphorylation on tumor growth and vascular endothelial growth factor expression in human gastric carcinoma. Pathobiology.

[46] Casalini P, Iorio MV, Galmozzi E, Ménard S (2004). Role of HER receptors family in development and differentiation. J Cell Physiol.

[47] Iida M, Brand TM, Starr MM, Huppert EJ, Luthar N, Bahrar H (2014). Overcoming acquired resistance to cetuximab by dual targeting HER family receptors with antibody-based therapy. Mol Cancer.

[48] Wheeler DL, Huang S, Kruser TJ, Nechrebecki MM, Armstrong EA, Benavente S (2008). Mechanisms of acquired resistance to cetuximab: role of HER (ErbB) family members. Oncogene.

[49] Hirata A, Hosoi F, Miyagawa M, Ueda S, Naito S (2005). HER2 overexpression increases sensitivity to gefitinib, an epidermal growth factor receptor tyrosine kinase inhibitor, through inhibition of HER2/HER3 heterodimer formation in lung cancer cells. Cancer Res.

[50] Huang S, Houghton PJ (2002). Inhibitors of mammalian target of rapamycin as novel antitumor agents: from bench to clinic. Curr Opin Investig Drugs.

[51] Motzer RJ, Escudier B, Oudard S, Hutson TE, Porta C, Bracarda S (2008). Efficacy of everolimus in advanced renal cell carcinoma: a double-blind, randomised, placebo-controlled phase III trial. Lancet.

[52] Zhang D, Sliwkowski MX, Mark M, Frantz G, Akita R, Sun Y (1997). Neuregulin-3 (NRG3): a novel neural tissue-enriched protein that binds and activates ErbB4. Proc Natl Acad Sci U S A.

[53] Harari D, Tzahar E, Romano J, Shelly M, Pierce JH, Andrews GC (1999). Neuregulin-4: a novel growth factor that acts through the ErbB-4 receptor tyrosine kinase. Oncogene.

[54] Hamburger AW (2008). The role of ErbB3 and its binding partners in breast cancer progression and resistance to hormone and tyrosine kinase directed therapies. J Mammary Gland Biol Neoplasia.

[55] Liu Z, Ahn JY, Liu X, Ye K (2006). Ebp1 isoforms distinctively regulate cell survival and differentiation. Proc Natl Acad Sci U S A.

[56] Soltoff SP, Carraway KL, Prigent SA, Gullick WG, Cantley LC (1994). ErbB3 is involved in activation of phosphatidylinositol 3-kinase by epidermal growth factor. Mol Cell Biol.

[57] Prigent SA, Gullick WJ (1994). Identification of c-erbB-3 binding sites for phosphatidylinositol 3′-kinase and SHC using an EGF receptor/c-erbB-3 chimera. EMBO J.

[58] Collier TS, Diraviyam K, Monsey J, Shen W, Sept D, Bose R (2013). Carboxyl group footprinting mass spectrometry and molecular dynamics identify key interactions in the HER2-HER3 receptor tyrosine kinase interface. J Biol Chem.

[59] Ueno Y, Sakurai H, Tsunoda S, Choo MK, Matsuo M, Koizumi K (2008). Heregulin-induced activation of ErbB3 by EGFR tyrosine kinase activity promotes tumor growth and metastasis in melanoma cells. Int J Cancer.

[60] Engelman JA, Janne PA, Mermel C, Pearlberg J, Mukohara T, Fleet C (2005). ErbB-3 mediates phosphoinositide 3-kinase activity in gefitinib-sensitive non-small cell lung cancer cell lines. Proc Natl Acad Sci U S A.

[61] Soler M, Mancini F, Meca-Cortes O, Sanchez-Cid L, Rubio N, Lopez-Fernandez S (2009). HER3 is required for the maintenance of neuregulin-dependent and -independent attributes of malignant progression in prostate cancer cells. Int J Cancer.

[62] Green AR, Barros FF, Abdel-Fatah TM, Moseley P, Nolan CC, Durham AC (2014). HER2/HER3 heterodimers and p21 expression are capable of predicting adjuvant trastuzumab response in HER2+ breast cancer. Breast Cancer Res Treat.

