# Supplementary figures and images for: Positive prognostic value of HER2-HER3 co-expression and p-mTOR in gastric cancer patients
Source: BMC Cancer. 2017 Dec 12;17:841. doi: 10.1186/s12885-017-3851-y (PMC5727869; doi:10.1186/s12885-017-3851-y)

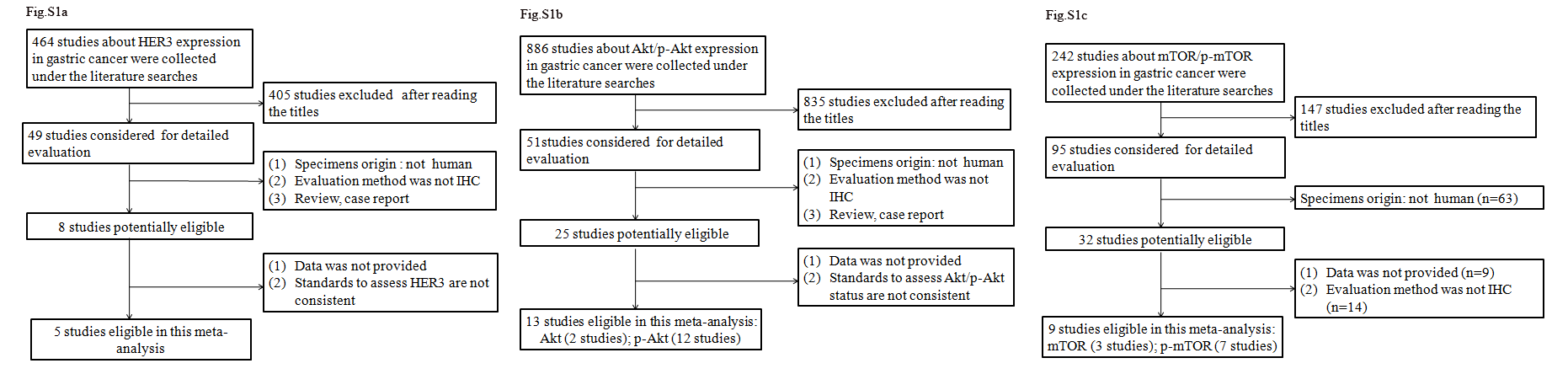

Supplement: Supplementary file 2 — Flow diagrams of study selection procedure. (TIFF 590 kb) [file 12885_2017_3851_MOESM2_ESM.tif]
