# Supplementary material for: Positive prognostic value of HER2-HER3 co-expression and p-mTOR in gastric cancer patients
Source: BMC Cancer. 2017 Dec 12;17:841. doi: 10.1186/s12885-017-3851-y (PMC5727869; doi:10.1186/s12885-017-3851-y)
Supplement: Supplementary file 3 — Clinicopathological parameters and quality scores of studies of each target protein in patients with gastric cancer. (DOCX 39 kb) [file 12885_2017_3851_MOESM3_ESM.docx]

**Table S1.** Clinicopathological parameters and quality scores of studies comparing HER3 positive GC with HER3 negative GC

| Studies | Number of Patients | Sex (male/female) | Depth of invasion (T1+T2/T3+T4) | LN metastasis (positive/negative) | Distant metastasis (M1/M0) | TNM stage (I+II/III+IV) | Quality  score |
| --- | --- | --- | --- | --- | --- | --- | --- |
| This study 2016 | 120(65 *vs*.55) | 42/23 38/17 | 7/58 15/40 | 54/11 41/14 | 3/62 3/52 | 17/48 16/39 | NA |
| Hayashi 2008 | 134(79 vs.55) | 51/28 43/12 | NA | 58/21 14/41 | 15/64 2/53 | NA | 8^a^ |
| Zhang 2009 | 102(14 vs.88) | NA | NA | NA | NA | 3/11 9/39 | 5^a^ |
| Wu 2014 | 161(90 vs.71) | 73/17 51/20 | 19/71 30/41 | 59/31 34/37 | 9/81 3/68 | NA | 5^a^ |
| Tang 2015 | 121(75 vs.46) | 52/23 33/13 | 8/67 3/43 | 58/17 32/14 | 5/70 4/42 | 23/52 13/33 | 5^a^ |
| He 2015 | 498(103vs.395) | 70/33 280/115 | 13/90 109/286 | 80/23 261/134 | 17/86 73/322 | 26/77 180/215 | 8^a^ |

**Table S2.** Clinicopathological parameters and quality scores of studies comparing Akt positive GC with Akt negative GC

| Studies | Number of Patients | Sex  (male/female)  Akt(+) Akt(-) | Age  (<60/>60)  Akt(+) Akt(-) | Tumor location (upper/low)  Akt(+) Akt(-) | Differentiation (well+moderate/poor)  Akt(+) Akt(-) | Depth of invasion (T1+T2/T3+T4)  Akt(+) Akt(-) | LN metastasis (positive/negative)  Akt(+) Akt(-) | Metastasis (positive/negative)  Akt(+) Akt(-) | Tumor stage (I+II/III+IV)  Akt(+) Akt(-) | Quality  score |
| --- | --- | --- | --- | --- | --- | --- | --- | --- | --- | --- |
| This study 2016 | 120(80*vs*.40) | 55/25 25/15 | 25/55 24/16 | 46/34 31/9 | 69/11 33/7 | 14/66 8/32 | 62/18 33/7 | 3/77 3/37 | 21/59 12/28 | NA |
| Nam 2003 | 311(230*vs*.81) | 151/79 56/25 | NA | 108/122 37/44 | NA | NA | 140/90 52/29 | 9/221 6/75 | 153/77 44/37 | 7^a^ |
| Gu 2014 | 70(26*vs*.44) | 18/8 30/14 | 18/8 22/22 | NA | 7/19 25/19 | NA | 25/1 30/14 | 8/18 4/40 | 2/24 12/32 | 7^a^ |

**Table S3.** Clinicopathological parameters and quality scores of studies comparing p-Akt positive GC with p-Akt negative GC

| Studies | Number of Patients | Sex  (male/female)  p-Akt(+) p-Akt (-) | Age  (younger/older)  p-Akt(+) p-Akt (-) | Tumor location (upper/low)  p-Akt(+) p-Akt (-) | Differentiation (well+moderate/poor)  p-Akt(+) p-Akt (-) | Depth of invasion (T1+T2/T3+T4)  p-Akt(+) p-Akt (-) | LN metastasis (positive/negative)  p-Akt(+) p-Akt (-) | Metastasis (positive/negative)  p-Akt(+) p-Akt (-) | Tumor stage  (I+II/III+IV)  p-Akt(+) p-Akt (-) | Quality  score |
| --- | --- | --- | --- | --- | --- | --- | --- | --- | --- | --- |
| This study 2016 | 120(71*vs*.49) | 46/25 34/15 | 28/43 21/28 | 46/25 31/18 | 59/12 43/6 | 9/62 13/36 | 59/12 36/13 | 1/70 5/44 | 20/51 13/36 | NA |
| Sasaki 2013 | 40(20*vs*.20) | NA | NA | NA | NA | 4/16 7/13 | 14/6 13/7 | NA | 7/13 9/11 | 7^a^ |
| Oki 2005 | 76(22*vs*.54) | 16/6 37/17 | NA | NA | 12/10 20/33* | NA | 12/10 28/26 | NA | 12/10 24/30 | 7^a^ |
| Nam 2003 | 311(242*vs*.69) | 165/77 45/24 | NA | 109/133 35/34 | NA | NA | 140/102 52/17 | 11/231 5/64 | 160/82 36/33 | 7^a^ |
| Han 2008 | 101(89*vs*.12) | NA | NA | NA | 51/38 10/2 | NA | NA | 43/46 9/3 | 42/47 7/5 | 7^a^ |
| Hisamatsu 2016 | 202(69*vs*.133) | 51/18 84/49 | NA | NA | NA | NA | 49/20 89/44 | NA | 34/35 54/79 | 8^a^ |
| Bian 2015 | 396(221*vs*.175) | 154/67 107/68 | 98/123 79/96 | 107/112 76/95 | NA | 69/152 63/112 | 148/73 84/91 | 25/196 9/166 | 94/127 105/70 | 7^a^ |
| Murakami 2007 | 140(81*vs*.59) | 49/32 35/24 | NA | NA | 40/41 36/23 | 28/53 20/39 | 47/34 31/28 | NA | 27/54 20/39 | 7^a^ |
| Chang 2015 | 424(151*vs*.273) | NA | 79/72 129/144 | NA | 73/78 147/126 | 91/60 128/145 | 63/88 147/126 | 5/146 7/265 | NA | 7^a^ |
| Sangawa 2014 | 75(30*vs*.45) | 24/6 29/16 | 3/27 7/38 | 20/10 30/15 | NA | 5/25 12/33 | 23/7 28/17 | NA | 3/27 8/37 | 7^a^ |
| Zhou 2012 | 48(32*vs*.16) | 18/14 10/6 | NA | NA | 20/12 14/2 | NA | 22/10 7/9 | NA | 10/22 12/4 | 7^a^ |
| Murayama 2009 | 109(94*vs*.15) | 68/26 9/6 | NA | NA | NA | NA | 47/47 6/9 | NA | NA | 8^a^ |
| Kobayashi 2006 | 88(50*vs*.38) | NA | NA | NA | 34/16 15/23 | 25/25 24/14 | 25/15 16/22 | NA | 26/24 24/14 | 8^a^ |

**Table S4.** Clinicopathological parameters and quality scores of studies comparing mTOR positive GC with mTOR negative GC

| Studies | Number of Patients | Sex  (male/female)  mTOR(+) mTOR(-) | Age  (<60/>60)  mTOR(+) mTOR(-) | Tumor location (upper/low)  mTOR(+) mTOR(-) | Differentiation (well+moderate/poor)  mTOR(+) mTOR(-) | Depth of invasion (T1+T2/T3+T4)  mTOR(+) mTOR(-) | LN metastasis (positive/negative)  mTOR(+) mTOR(-) | Metastasis (positive/negative)  mTOR(+) mTOR(-) | Tumor stage (I+II/III+IV)  mTOR(+) mTOR(-) | Quality  score |
| --- | --- | --- | --- | --- | --- | --- | --- | --- | --- | --- |
| This study 2016 | 120(73*vs*.47) | 50/23 30/17 | 21/52 28/19 | 49/24 28/19 | 60/13 42/5 | 10/63 12/35 | 61/12 34/13 | 3/70 3/44 | 15/58 18/29 | NA |
| Li 2012 | 33(17*vs*.16) | 8/9 7/9 | NA | NA | 4/13 11/5 | NA | 13/4 3/13 | NA | 3/14 15/1 | 7^a^ |
| Yu 2009 | 1072(545*vs*.527) | 395/150 362/165 | 266/279 271/256 | 276/248233/271* | 370/175 264/263 | 175/370 137/390 | 185/360 171/356 | NA | 224/321 203/324 | 9^a^ |
| Xi ao 2009 | 412(255*vs*.157) | 179/76 109/48 | NA | NA | NA | 143/112 79/78 | 173/82 90/67 | NA | NA | 8^a^ |

**Table S5.** Clinicopathological parameters and quality scores of studies comparing p-mTOR positive GC with p-mTOR negative GC

| Studies | Number of Patients | Sex  (male/female)  p-mTOR(+) p-mTOR (-) | Age  (<60/>60)  p-mTOR(+) p-mTOR (-) | Tumor location (upper+medium/low)  p-mTOR(+) p-mTOR (-) | Differentiation (well+moderate/poor)  p-mTOR(+) p-mTOR (-) | Depth of invasion (T1+T2/T3+T4)  p-mTOR(+) p-mTOR (-) | LN metastasis (positive/negative)  p-mTOR(+) p-mTOR (-) | Metastasis (positive/negative)  p-mTOR(+) p-mTOR (-) | Tumor stage (early/anvanced)  p-mTOR(+) p-mTOR (-) | Quality  score |
| --- | --- | --- | --- | --- | --- | --- | --- | --- | --- | --- |
| This study 2016 | 120(65*vs*.55) | 46/19 34/21 | 26/39 23/32 | 43/22 34/21 | 49/16 53/2 | 6/59 16/39 | 56/9 39/16 | 2/63 4/51 | 12/53 21/34 | NA |
| Xu 2010 | 181(93*vs*.88) | 66/27 63/25 | 45/48 50/38 | 62/31 43/43* | 30/63 29/59 | 22/71 30/58 | 78/15 59/29 | NA | 28/65 45/43 | 8^a^ |
| Bian 2015 | 396(293*vs*.103) | 196/97 65/38 | 121/172 56/47 | 135/154 48/53* | NA | 88/205 44/59 | 187/106 45/58 | NA | 133/160 66/37 | 7^a^ |
| Inokuchi 2011 | 126(81*vs*.45) | 57/24 31/14 | NA | NA | NA | NA | 49/32 18/27 | 28/53 10/35 | 35/46 28/17 | 7^a^ |
| Yu 2009 | 1072(499*vs*.573) | 345/154 412/161 | 217/282 320/253 | 96/382 84/466* | 307/192 327/246 | 135/346 177/396 | 132/367 224/349 | NA | 172/327 255/318 | 9^a^ |
| An 2010 | 290(131*vs*.159) | 88/43 97/62 | NA | 65/63 77/79* | 52/79 43/116 | NA | 35/96 61/98 | NA | NA | 9^a^ |
| Murayama 2009 | 109(69*vs*.40) | 51/18 26/14 | NA | NA | NA | NA | 40/29 13/27 | NA | NA | 8^a^ |
| Byeon 2014 | 700(221vs.479) | 158/63 318/161 | 110/111 258/221 | NA | NA | NA | NA | NA | 116/105 259/220 | 7^a^ |

GC: gastric cancer; NA: not available

TNM stages are based on tumor-node-metastasis classification advocated by International Union against Cancer

Quality score^a^: use the Newcastle-Ottawa scale (stars)
