# Supplementary material for: Positive prognostic value of HER2-HER3 co-expression and p-mTOR in gastric cancer patients
Source: BMC Cancer. 2017 Dec 12;17:841. doi: 10.1186/s12885-017-3851-y (PMC5727869; doi:10.1186/s12885-017-3851-y)
Supplement: Supplementary file 1 — Certification of Ethics. (DOCX 219 kb) [file 12885_2017_3851_MOESM1_ESM.docx]

**Letter to the editor**

Dear editor,

Thanks for your advises and we are pleased to accomplish the relevant tasks. However, we only have the ethical demonstration of Chinese version, and we are applying for an English version of ethical demonstration, and will get it at the end of this month. So we submit the manuscript again, and provide the Chinese version and its English translation. Please contact me if any question existed.

Sincerely yours,

Mao-Ming Xiong

Department of General Surgery,

The First Affiliated Hospital of Anhui Medical University, Anhui Medical University,

218 Jixi Road, Hefei 230022, Anhui Province, China

E-mail: ayfyxmm@163.com

**(English Translation)**

**Approved Code:20150073**

**ANHUI MEDICAL UNIVERSITY**

**Verification of Biomedical Research Ethics Committee**

**(Formal Version)**

**Principal Investigator: Bo Chen**

**Project Name:** The molecular mechanism of enhanced activity of HIF-1α/PKM2 signaling pathway in Helicobacter pylori-associated gastric carcinogenesis

**Institution of Applicant: Anhui Medical University**

1. The bio-medical ethics committee have demonstrated the program of this project, and cautiously discussed the following three points:

①: The rights and interests of the research objects;

②: The measures to obtain the right of knowing and consent;

③: The potential danger and possible benefit.

2. Approved to perform this research plan. In this project implementation process, informed consent form, questionnaire	and relevant instruction should be used.

3. If the project program needs to be revised, it must be demonstrated by the bio-medical ethics committee in advance. At the same time, the modification details and reasons should be recorded.

4. If any adverse reactions appeared, submit a written report to the bio-medical ethics committee.

The Chairman’s Name: Qi-xing Zhu	Signature:

Effective Date: 2015-03-01

Biomedical Research Ethics Committee of Anhui Medical University
